# The Pattern of Attrition from an Antiretroviral Treatment Program in Nigeria

**DOI:** 10.1371/journal.pone.0051254

**Published:** 2012-12-13

**Authors:** Solomon Odafe, Kwasi Torpey, Hadiza Khamofu, Obinna Ogbanufe, Edward A. Oladele, Oluwatosin Kuti, Oluwasanmi Adedokun, Titilope Badru, Emeka Okechukwu, Otto Chabikuli

**Affiliations:** 1 Prevention, Care and Treatment Department, Abuja, Nigeria; 2 Monitoring and Evaluation Department, Abuja, Nigeria; 3 HIV/AIDS/TB Unit USAID Nigeria, Abuja, Nigeria; 4 Department of Family Medicine, Medical University of Southern Africa, Medusa, South Africa; 5 Southern Africa Region, Pretoria, South Africa; Vanderbilt University, United States of America

## Abstract

**Objective:**

To evaluate the rate and factors associated with attrition of patients receiving ART in tertiary and secondary hospitals in Nigeria.

**Methods and Findings:**

We reviewed patient level data collected between 2007 and 2010 from 11 hospitals across Nigeria. Kaplan-Meier product-limit and Cox regression were used to determine probability of retention in care and risk factors for attrition respectively. Of 6,408 patients in the cohort, 3,839 (59.9%) were females, median age of study population was 33years (IQR: 27–40) and 4,415 (69%) were from secondary health facilities. The NRTI backbone was Stavudine (D4T) in 3708 (57.9%) and Zidovudine (ZDV) in 2613 (40.8%) of patients. Patients lost to follow up accounted for 62.7% of all attrition followed by treatment stops (25.3%) and deaths (12.0%). Attrition was 14.1 (N = 624) and 15.1% (N = 300) in secondary and tertiary hospitals respectively (p = 0.169) in the first 12 months on follow up. During the 13 to 24 months follow up period, attrition was 10.7% (N = 407) and 19.6% (N = 332) in secondary and tertiary facilities respectively (p<0.001). Median time to lost to follow up was 11.1 (IQR: 6.1 to 18.5) months in secondary compared with 13.6 (IQR: 9.9 to 17.0) months in tertiary sites (p = 0.002). At 24 months follow up, male gender [AHR 1.18, 95% CI: 1.01–1.37, P = 0.038]; WHO clinical stage III [AHR 1.30, 95%CI: 1.03–1.66, P = 0.03] and clinical stage IV [AHR 1.90, 95%CI: 1.20–3.02, p = 0.007] and care in a tertiary hospital [AHR 2.21, 95% CI: 1.83–2.67, p<0.001], were associated with attrition.

**Conclusion:**

Attrition could potentially be reduced by decentralizing patients on ART after the first 12 months on therapy to lower level facilities, earlier initiation on treatment and strengthening adherence counseling amongst males.

## Introduction

The last decade has seen a rapid scale up of ART programs in developing countries largely supported by the WHO's ‘3 by 5’ initiative and the U.S President's Emergency Plan for AIDS Relief (PEPFAR). While the success of ART scale up has been widely acknowledged, retaining patients in care remains a well-documented challenge globally [1], [2]. Retention is defined as the proportion of patients alive and receiving ART after a defined follow up period [3]. Patients' retention is a function of attrition which includes deaths, patients lost to follow up and those who stopped treatment [3], [4]. Most patient attrition occur within the first year on ART and patient retention across low and middle income countries in 2009 was estimated at 82% after 12 months on ART [1]. Rosen et al in a meta-analysis in 2010 showed a retention rate of 86% at 6 months and 76% at the end of the year 2 [5]. A combination of social, economic and structural factors contribute to attrition in ART programs in sub-Saharan Africa; they include formal and informal costs, poverty, and adverse effects of drugs, nondisclosure, long waiting times, alcohol abuse, and use of traditional medicines [6], [7], [8].

Nigeria, with about 2% of the world's population accounts for about 10% of PLHIV globally with an estimated at 3.3 million PLHIV [9]. Of these, about a million need ART; the government with support of several partners has rapidly scaled up ART enrolment and has steadily increased number of patients initiated on ART from 90,008 in 2006 [10] to an estimated 300,000 at the end of 2009 [11]. Initiation of PLHIV on ART in the country is currently restricted to secondary and tertiary level hospitals. However to improve access to ART, the government plans to decentralize services to primary health centers [12]. There are concerns that this will increase attrition because documented challenges of delivering ART in low resource settings such as shortages of health care staff, weak clinical care and diagnostic capacity and poor data management systems [13], [14], [15] are more likely in lower levels of care.

A previous study in Nigeria, compared treatment outcomes in secondary and tertiary ART centers in adults' patients [16]. This study compared the rates of attrition from care in tertiary and secondary health facilities in adults and children and examined effects of antiretroviral drugs (ARVs) and other clinical factors on attrition.

## Materials and Methods

### Study design and setting

This is a retrospective cohort study that reviewed patient level data collected between 2007 and 2010. The levels of care in the public sector in Nigeria are primary health centers, typically staffed by nurses, community health officers (CHOs), community health extension workers (CHEWs), junior CHEWs and environmental health officers; secondary level hospitals, typically staffed by medical officers, nurses, midwives, laboratory scientists, pharmacists and community health officers; and tertiary centers, typically staffed by medical specialists [17]. Commencement of PLHIV on ART in Nigeria is mainly in Government-owned secondary and tertiary level hospitals.

The Global HIV/AIDS Initiative Nigeria (GHAIN) was funded by the President's Emergency Plan for AIDS Relief through United States Agency for International Development. The project provides technical assistance to selected health facilities that offer comprehensive HIV/AIDS services Nigeria between 2004 and 2011. In 2007 Family Health International (FHI360) introduced the Lafiya Management Information System (LAMIS®), an open source electronic medical records (EMR) system in eleven hospitals in Nigeria. They include four tertiary and seven secondary hospitals. Patient monitoring and management in the selected hospitals were based on Nigerian national ART guidelines, which were adapted from the WHO guidelines [18]. The LAMIS was used to produce reports on patients who defaulted from clinical appointment. These patients were tracked using their home addresses and telephone numbers collected during registration. Tracking outcomes were updated regularly into the LAMIS®.

### Inclusion criteria

Eleven hospitals providing adult and pediatric ART services and using LAMIS since 2007 were included in the study. A cohort of patients (adults and children) who were initiated on ART between January 1^st^ 2007 and December 31^st^ 2007 at these hospitals were followed up for 24 months. Table 1 shows list of facilities included in the study.

**Table 1:** Selected health care facilities.

Type facility	Name	State	Region in Nigeria
**Tertiary**	Federal Medical Center (FMC) Owo	Ondo	South-West
	FMC Owerri	Enugu	South-East
	FMC Jalingo	Taraba	North-East
	FMC Yola	Adamawa	North-East
**Secondary**	Maitama District Hospital	Abuja	North-Central
	Infectious Disease hospital, Kano	Kano	North-West
	Murtala Mohammed Specialist Hospital, Kano	Kano	North-West
	Mainland Hospital Yaba	Lagos	South-West
	Calabar General Hospital	Cross River	South-South
	Lawrence Henshaw Memorial Hospital, Calabar	Cross River	South-South
	Massey Street Children's Hospital	Lagos	South-West

### Patient monitoring and management

Patients were assessed for ART eligibility based on CD4 count and WHO clinical staging. Adults, adolescents and children >5 years were considered eligible for ART when their CD4 count was <200 cells/mm^3^ or they present with WHO stage IV [19], [20]. Children between 12 and 35 months were considered eligible for ART when they present with a CD4% of <20% (750 cells/mm^3^) or WHO stage III or IV disease. While children between 36 and 59 months were considered eligible for ART when they present with a CD4% of <15% (350 cells/mm^3^) or WHO stage III or IV disease [19]. After assessment for ART eligibility, patients were required to go through three sessions of adherence counseling and identify a treatment supporter to help with adherence. Patient or guardian (or next of kin) of pediatric patients were required to attend the adherence counseling sessions before ART initiation. Patients and parents/guardians undergo group and individual counseling sessions and are educated on basic facts on HIV infection, antiretroviral drugs, their side effects and the implications of ART. Once started on ART, patients are reviewed at the HIV/AIDS clinic after 2 weeks. From then on, provided there are no adverse drug reactions, patients are seen and receive drug refills at 4 week intervals for the first 6 months. Thereafter they are seen at three to six monthly intervals. Patients receive on-going adherence counseling by a counselor at every contact with the hospital. In addition patients see pharmacist at the point of dispensing who provides drug information and reinforces adherence counseling. The first-line ART regimen at time of study was a fixed-dose combination of Zidovudine (AZT), lamivudine (3TC) and Nevirapine (NVP). In case of AZT- and NVP- related toxicity, the respective alternatives were Stavudine (d4T) or Tenofovir (TDF) and Efavirenz (EFV).

### Data collection

Data collected during routine clinical visits using standardized national tools, which include ART care cards, pharmacy and laboratory order forms, Pre-ART and ART registers were entered into LAMIS®. The data entry at the site was carried out by clerks that were trained on the tools and supervised by record staff to ensure data were appropriately captured into the EMR. Electronic processes are also available in the EMR that calls attention of operators to inconsistencies during data entry. LAMIS® data from these sites were transmitted securely to a central database and exported to STATA® version 10. The export process in LAMIS® automatically scrambles all patients' personal identifiers.

### Variables

The outcome variable was attrition from care. Attrition in this study refers to the patients who stopped treatment, or were confirmed dead or lost to follow-up (LTFU). A patient was deemed to have ‘stopped treatment’ when s/he stopped ART for any reason including medico-social reasons [21]; those LTFU were patients who had been absent from treatment for at least 90 days from the last given appointment date and at least three attempts at tracking failed; while ‘dead’ patients were those known to have died. Patients classified as dead were those who died in the hospital and appropriately captured in hospital records or discovered to have died during tracking from next of kin or relations. [21]. Information on deaths was obtained from patient records or through contact of next of kin or relatives during tracking. Explanatory variables considered were patients' age, sex, CD4 count, WHO clinical stage and regimen at ART initiation.

### Data analysis

The total time of observation contributed by each patient was summed up to obtain the total person years of observation. Time on ART was calculated in months using the time interval between the date of ART initiation and (1) date of known death (2) date of leaving the program due to LTFU, or stopping of treatment and (3) date 24 months of follow-up had accrued. We calculated attrition rates as a proportion of patients lost from care through death, LTFU and those who stopped treatment at 12 and 24 months observation period. The Wilcoxon sign rank test was used to compare median changes in CD4 counts between patients in secondary and tertiary hospitals. Chi-square test was used to test the differences in proportions. The probability of retention in care was estimated using Kaplan-Meier product-limit method. Potential explanatory variables of attrition were checked with the Breslow-Gehan test. A multivariable analysis for attrition was conducted using Cox proportional hazard model. The incidence rates of subcategories of attrition (dead, LTFU and treatment stopped) were expressed as the number of patients with at least one occurrence of the given event per 100 person years. A two-tailed statistical analysis with a p-value<0.05 was considered to be statistically significant for all tests conducted.

### Ethics statement

This research analyzed retrospectively routinely collected program data. There was no patient contact. However patients in respective hospitals were not required to provide written consent to access services or have their data stored but were informed during registration that data could be used for research. Ethical approval to use the stored data was obtained from the Nigerian National Health and Research Ethics Committee (approval number NHREC/01/01/2007-24/09/09c) and FHI's Protection of Human Subjects Committee (PHSC). Study was determined to be exempt from oversight after review by both committees. Data entry clerks were trained on confidentiality and secure data transmission. Patient personal identifiers were scrambled at the point of data export.

## Results

### Baseline characteristics

A total of 6,417 patients started ART between January and December 2007. Of these, 9 patients were excluded from the analysis due to incomplete data. Of the 6,408 patients, 4,415 (69.0%) and 1,993 (31.0%) patients commenced ART at secondary and tertiary facilities respectively. Female patients made up 59.9% (3,839) of the study population and 94.4% (6,046) were aged 15 and above. A substantial proportion of patients were initiated on ART in WHO stage II (39.6%) and III (39.7%). The vast majority of patients were on Stavudine-based regimen (58%). Table 2 summarizes baseline characteristics of study population.

**Table 2:** Baseline characteristics of the Study population.

*Variable*	*All facilities*	*Tertiary hospital*	*Secondary hospital*	*P-value*
	*n (%)*	*n (%)*	*n (%)*	
Total	6408	1993	4415	
**Gender**				
Male	2569 (40.1)	797 (40.0)	1772 (40.0)	
Female	3839 (59.9)	1196 (60.0)	2643 (60.0)	0.912
**Age group (years)**				
<15	362 (5.6)	105 (5.3)	257 (5.8)	
≥15	6046 (94.4)	1888 (94.7)	4158 (94.2)	0.375
Median (IQR)	33 (27–40)	33 (27–41)	33 (27–40)	
**WHO clinical stage** *				
Stage I	1126 (17.9)	321(16.7)	805 (18.5)	
Stage II	2482 (39.6)	1298 (67.7)	1184 (27.2)	
Stage III	2490 (39.7)	266 (13.9)	2224 (51.1)	
Stage IV	173 (2.8)	32 (1.7)	141 (3.2)	<0.001
**Type of Regimen**				
AZT based	2613 (40.8)	360 (18.0)	2253 (51.0)	
d4T based	3708 (57.8)	1616 (81.2	2092 (47.4)	
TDF based	81 (1.3)	14 (0.7)	67 (1.5)	<0.001
Others	6(0.1)	3 (0.2)	3 (0.1)	

* Data available for 6,271 (Tertiary: 1917, Secondary: 4354).

### Attrition at 12 and 24 months

Patients were followed up for 11,038 person years. Tertiary and secondary facilities contributed 3,348 and 7,690 person-years of follow up respectively. Attrition from all facility types at the first 12 months on ART was 924 (14.5%). In all facilities at the first 12 months, LTFU accounted for 8.2% (N = 524), deaths 2.4% (N = 153) and treatment stopped 3.9% (N = 247). Attrition was 14.1 (N = 624) and 15.1% (N = 300) in secondary and tertiary hospitals respectively (p = 0.169) in the first 12 months on follow up. Tables 3 show attrition from care and proportions in the first 12 months follow up period.

**Table 3:** Treatment outcomes for all patients on ART at tertiary and secondary health facilities at 0–12 months follow up.

	*Total*		*Secondary*		*Tertiary*		*P-value*
	n (%)	Rate/100py	n (%)	Rate/100py	n (%)	Rate/100py	
Total started on ART	6408		4415		1993		
Lost to follow up	524 (8.2)	8.5	386 (8.7)	9.5	138 (6.9)	7.4	<0.001
Dead	153 (2.4)	2.2	130 (2.9)	3.2	23 (1.2)	1.2	<0.001
Treatment stopped	247 (3.9)	5.1	108 (2.4)	2.7	139 (7.0)	7.5	<0.001
Total attrition	924 (14.5)		624 (14.1)		300 (15.1)		0.169
Retained in care	5484 (85.5)		3791(85.9)		1693 (84.9)		0.322

In the 13 to 24 months follow up period, total attrition from all facility type was 739 (13.5%), LTFU accounted for 9.4% (N = 518), deaths 0.9% (N = 47) and treatment stopped 3.2% (N = 174) of patients on ART. Attrition of patients on ART in secondary and tertiary facilities during the 13 to 24 months follow up period was 10.7% (N = 407) and 19.6% (N = 332) respectively (p<0.001). Tables 4 show attrition from care and proportions in the 13 to 24 months follow up period.

**Table 4:** Treatment outcomes for all patients on ART at tertiary and secondary health facilities at 13–24 months follow up.

	*Total*		*Secondary*		*Tertiary*		*P-value*
	n (%)	Rate/100py	n (%)	Rate/100py	n (%)	Rate/100py	
Total started on ART	5484		3791		1693		
Lost to follow up	518 (9.4)	9.8	303 (8.0)	9.0	215 (12.7)	10.5	<0.001
Dead	47 (0.9)	0.9	44 (1.2)	2.3	3 (0.2)	0.8	<0.001
Treatment stopped	174 (3.2)	3.2	60 (1.6)	2.2	114 (6.7)	7.6	<0.001
Total attrition	739 (13.5)		407 (10.7)		332 (19.6)		<0.001
Retained in care	4745 (86.5)		3384 (89.3)		1361(80.4)		<0.001

**Figure 1:**
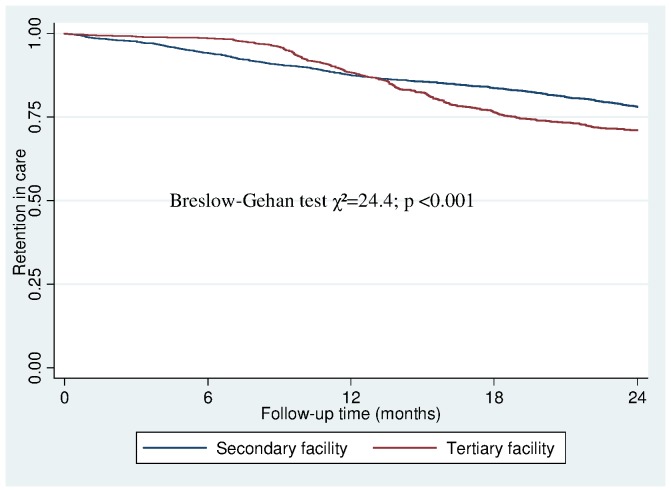
Kaplan-Meier curve showing retention in care for 24 months in secondary and tertiary facilities. (Breslow-Gehan test χ^2^ = 24.4; p<0.001).

Median time to LTFU for all facilities was 12.0 months (IQR: 7.3–18.0) while median time to death was 4.3 months (IQR: 1.2–11.6). There was statistically significant difference in median time from ART start to attrition for both facility types. Median time to LTFU was 11.1 months (IQR: 6.1–18.5) and 13.6 months (IQR: 9.9–17.0) in secondary and tertiary facilities respectively (p = 0.002). The median time to death was 5.0 months (IQR: 1.3–12.0) and 2.4 months (IQR: 0.2–6.6) in secondary and tertiary facilities respectively (p = 0.032). The Kaplan-Meier estimates for the probability of attrition in all facility types at 3, 6, 12 and 18 months were 0.02, 0.04, 0.12 and 0.18 respectively. Figure 1 shows differences in Kaplan-Meier curves for retention in secondary and tertiary facilities.

### Risk factors for attrition

Type of health facility was associated with the risk of attrition in the early and later time periods on ART. Patients receiving treatment at tertiary health facilities had a lower risk of attrition than patients in secondary facilities during the first 12 months of follow-up [HR 0.61, 95% CI: 0.51 to 0.73, p<0.001]. On the contrary, patients receiving treatment at tertiary health facilities were at much higher risk of attrition than patients in secondary facilities in the second year of follow-up [HR 2.21, 95% CI:1.83 to 2.67, p<0.001]. Risk of attrition increased with increasing WHO clinical stage in the second year of follow-up; Stage II [HR 1.32, 95% CI: 1.05 to 1.66, p = 0.016], Stage III [HR 1.30, 95% CI: 1.03 to 1.65, p = 0.030] and Stage IV [HR 1.90, 95% CI: 1.20 to 3.02, p = 0.007]. During the second year of follow-up, male patients had a higher risk of attrition compared with females [HR 1.18, 95% CI: 1.01 to 1.37, p = 0.038]. Patients on Tenofovir [HR 7.15, 95% CI: 2.61–19.57, p<0.001 and Stavudine [HR 1.19, 95% CI: 1.03–1.37] based regimen] had a higher risk of attrition compared with patients on Zidovudine based regimen during the first 12 months of follow-up. On the contrary, patients on Tenofovir [HR 0.30, 95% CI: 0.10–0.93, p = 0.038 and Stavudine [HR 0.76, 95% CI: 0.63–0.91] based regimen had 70% and 30% reduction in the risk of attrition respectively compared with patients on Zidovudine based regimen during the second year of follow-up. Table 5 summarizes the proportional hazards of baseline characteristics associated with attrition.

**Table 5:** Cox Proportional Hazard Models between Attrition and Predictor Variables: 0–12 and 13–24 months on ART.

Variable	0–12 months				13–24 months			
	Univariate		Multivariate		Univariate		Multivariate	
	HR (95% C.I)	P-value	HR (95% C.I)	P-value	HR (95% C.I)	P-value	HR (95% C.I)	P-value
**Age (years)**								
<15	1		1		1		1	
≥15	0.84(0.64–1.12)	0.235	0.85(0.63–1.16)	0.307	0.82 (0.61–1.11)	0.197	0.91 (0.66–1.27)	0.597
**Gender**								
Female	1		1		1		1	
Male	1.12 (0.98–1.29)	0.098	1.12(0.97–1.29)	0.122	1.20 (1.03–1.40)	0.017	1.18(1.01–1.37)	0.038
**Type of health facility**								
Secondary	1		1		1		1	
Tertiary	0.59 (0.51–0.68)	<0.001	0.61 (0.51–0.73)	<0.001	1.93 (1.66–2.25)	<0.001	2.21 (1.83–2.67)	<0.001
**WHO clinical stage**								
Stage I	1		1		1		1	
Stage II	0.87 (0.68–1.11)	0.260	1.02 (0.79–1.32)	0.889	1.50 (1.20–1.87)	<0.001	1.32(1.05–1.66)	0.016
Stage III	1.29 (1.01–1.64)	0.039	1.20(0.93–1.53)	0.159	1.13 (0.90–1.43)	0.285	1.30(1.03–1.66)	0.030
Stage IV	1.65 (1.14–2.39)	0.008	1.53(1.05–2.23)	0.028	1.77 (1.12–2.81)	0.015	1.90(1.20–3.02)	0.007
**CD4 count**								
<200	1		1		1		1	
200–350	0.90 (0.76–1.07)	0.235	0.96(0.81–1.15)	0.672	0.95 (0.79–1.13)	0.539	1.01(0.84–1.20)	0.930
>350	0.98 (0.76–1.26)	0.871	1.13(1.03–1.37)	0.384	1.20 (0.94–1.52)	0.148	1.27(0.98–1.65)	0.070
**Type of Regimen**								
AZT based	1		1		1		1	
d4T based	1.10 (0.96–1.27)	0.161	1.19(1.03–1.37)	0.019	1.04 (0.89–1.21)	0.630	0.76 (0.63–0.91)	0.002
TDF based	4.72 (2.10–10.64)	<0.001	7.15(2.61–19.57)	<0.001	0.33 (0.10–1.01)	0.053	0.30(0.10–0.93)	0.038

## Discussion

Our observation of retention patterns in the two levels of patient care using Kaplan-Meier curves suggested that attrition was less in tertiary centers in the first 12 months but progressively became worse over the following 12 months. Judging from the higher proportion of patients with stage III and IV disease in secondary hospitals compared to tertiary sites, it is likely that higher early loses in the secondary hospitals may be due to higher proportion of patients with advance disease. Whereas losses in tertiary sites during the following 12 months period were due mainly to LTFU and treatment stop.

The higher proportions of patients on stavudine in tertiary (81.2% vs 47.4%) compared to secondary sites may play a role in high rates of treatment stop observed in tertiary sites. High use of stavudine containing regimen have been reported elsewhere in Africa, studies in Mozambique and Cote d'Ivoire, reported 88% and 73% respectively of their study population initiated on Stavudine based regimen [22], [23]. Previous studies have also reported significant toxicities and treatment stop with stavudine based regimen [23], [24], [25], [26], [27]. However during the 13 to 24 months follow up period, we observed that initiation on Tenofovir and Stavudine based regimen was associated with less LTFU and mortality than those on Zidovudine. One study suggested that incidence of mild to severe anemia associated with Zidovudine may be a factor [23]. However, another study in Nigeria reported that patients on Tenofovir and Zidovudine had better retention than those on Stavudine based regimen [28].

An interesting finding however was the relatively high rates of mortality and LTFU in the first 12 months amongst patients on TDF containing regimen compared with ZDV. The exact reason for this finding was unclear and will require further research. However, a study that investigated causes for discontinuing treatment amongst patients on TDF, reported that about 23.8% of patients on TDF in that study population discontinued therapy due mainly to adverse reaction [29]. Another study in South Africa reported that patients with preexisting mild to moderate renal dysfunction were at greatest risk of death [30]. The South African study, also found that majority of deaths associated with TDF occurred within the first year on ART [30].

Our study found that patients with WHO clinical stage III or IV and patients with CD4 count less than 200 cells/mm3 had a higher risk of attrition compared to other categories of patients. Previous studies also identified severe immunosuppression as a reason for attrition [27], [31], [32], [33]. Another predictor of attrition observed in our study was male gender, as reported in previous studies in Africa [3], [28]. This finding supports the gender differences in health seeking behaviors reported by some studies [28], [33], [34]. However, there seem to be conflicting reports on the effect of gender on health seeking behavior. Other studies in Africa could not establish a link between gender and service utilization [35].

LTFU accounted for 67.5% of all attrition from care from zero to 24 months; this observation was comparable with reports from previous studies in resource limited settings that identified LTFU as a major reason for attrition [3], [5], [28]. We did not investigate reasons for LTFU in our study population but; existing literature suggest that majority of patients reported as LTFU may have actually died [31], [32], [36], [37]. In the first 12 months of follow up, we observed higher proportion of LTFU in secondary hospitals amongst study population (8.7 vs 6.9), there was however a reversal of pattern in the next 12 months of follow up with tertiary sites now having more patients LTFU. The higher proportion of early LTFU in secondary sites can be attributed to the higher proportion of patients with advance immunosuppression observed in that facility type. With regards to median time to LTFU we observed that patients in tertiary centers had a longer median time (13.6 vs. 11.1 months) to LTFU than those in secondary hospitals. This finding suggests that although a greater proportion of patients were LTFU in tertiary sites, they were retained for a longer period before eventual LTFU.

Death rates observed in our study population ranged from 2.3 to 3.2 and 0.8 to 1.2 per 100 person-years in secondary and tertiary hospitals respectively were lower than findings from studies in similar settings that reported death rates ranging from 2.9 to 30.1 per 100 person years [28], [31]. The lower death rates observed in our study may be attributable to the lower proportion (15.6%) of patients with advance HIV disease (WHO stage III and IV) or poor documentation of deaths because we could not ascertain true status of patients LTFU in our study population. Throughout the follow up period there were higher proportions of deaths in secondary hospitals compare with tertiary sites. The higher proportion of deaths in secondary level hospitals may be due to the higher proportion (54.3%) of patients in WHO stage III and IV in secondary than in tertiary (15.6%).

Forty-one percent of deaths observed in our study, occurred within the first 3 months on ART; this observation was consistent with findings from several studies in resource constraint settings that reported majority of mortality occurring within the first few months on treatment [36], [37], [38], [39]. With respect to median time to death, we observed that deaths occurred about twice as fast in tertiary hospitals than secondary (2.4 vs. 5.0 months respectively). In our environment, patients who are critically ill or have received sub optimal treatment in other facilities usually resort to tertiary hospital as the last hope for treatment. This may explain the shorter time to death for patients who died in tertiary hospitals compared to secondary facilities.

Previous studies reviewing retention rates in children reported encouraging results. One study that analyzed retention rates amongst 1184 children on ART in eight countries in resource limited countries reported retention rates of 85% after 12 months [40]. Another study in children with a median age of 6.5 years on ART in Cote D'Ivoire, reported survival probabilities of 91%, 88% and 86% at 12, 36 and 48 months respectively [41]. However in our study, we did not find a statistically significant difference in attrition rates between patients aged ≥15 and those less than 15.

In our study, the composite measure used for attrition ensured that all parameters of attrition in the patient group were factored into analysis. This has an advantage of giving a clear snapshot of attrition compared to single-factor measures. Additionally, the large number of patients studied and the wide geographic spread make for reliable inferences. A limitation of our study was the lack of data identifying reasons for LTFU as this information was not routinely captured. Additionally the effect of patient load in secondary and tertiary sites was not factored into the multivariate analysis.

## Conclusion

The attrition profile of secondary hospitals seems on the long term to be better than that in tertiary hospitals despite tertiary facilities being better equipped, better financed and more staffed than secondary facilities. The likelihood of attrition also increased with duration on ART. Other factors associated with attrition included: male gender, age <15 years and WHO stage III and IV. Study findings suggest that attrition from care could be reduced by decentralizing ART services to lower health systems after 12 months on therapy, earlier initiation of treatment, and careful consideration of antiretroviral drug adverse reactions during regimen selection as well as strengthening adherence counseling amongst adult males. However systems in lower health care facilities need to be strengthened to reduce risk of mortality of patients at that level of care.
